# *Drosophila* vinculin is more harmful when hyperactive than absent, and can circumvent integrin to form adhesion complexes

**DOI:** 10.1242/jcs.189878

**Published:** 2016-12-01

**Authors:** Aidan P. Maartens, Jutta Wellmann, Emma Wictome, Benjamin Klapholz, Hannah Green, Nicholas H. Brown

**Affiliations:** Department of Physiology, Development and Neuroscience, and the Gurdon Institute, University of Cambridge, Downing St., Cambridge CB2 1DY, UK

**Keywords:** Adhesion, *Drosophila*, Vinculin, Integrin, Talin, Rhea, Protein complex

## Abstract

Vinculin is a highly conserved protein involved in cell adhesion and mechanotransduction, and both gain and loss of its activity causes defective cell behaviour. Here, we examine how altering vinculin activity perturbs integrin function within the context of *Drosophila* development. Whereas loss of vinculin produced relatively minor phenotypes, gain of vinculin activity, through a loss of head–tail autoinhibition, caused lethality. The minimal domain capable of inducing lethality is the talin-binding D1 domain, and this appears to require talin-binding activity, as lethality was suppressed by competition with single vinculin-binding sites from talin. Activated *Drosophila* vinculin triggered the formation of cytoplasmic adhesion complexes through the rod of talin, but independently of integrin. These complexes contain a subset of adhesion proteins but no longer link the membrane to actin. The negative effects of hyperactive vinculin were segregated into morphogenetic defects caused by its whole head domain and lethality caused by its D1 domain. These findings demonstrate the crucial importance of the tight control of the activity of vinculin.

## Abstract (precis)

**Summary:** Development is more sensitive to gain of vinculin activity than its loss, and vinculin can promote cytoplasmic adhesion complexes independently of the usual integrin cue.

## INTRODUCTION

Cell adhesion is mediated by multiprotein complexes that link transmembrane receptors to the cytoskeleton. These complexes are assembled at discrete sites of the membrane, and both loss and gain of adhesion protein activity causes cellular and developmental defects (Wickstrom et al., 2011; Maartens and Brown, 2015a), which have pathological consequences (Winograd-Katz et al., 2014).

The first step in building a cell–matrix adhesion is the binding of transmembrane integrin receptors to extracellular matrix (ECM) components. This is followed by recruitment of cytoplasmic adhesion proteins, for example talin (also known as Rhea in flies), which occurs through the cytoplasmic tail of integrin. Talin is a crucial component of the link as it can simultaneously bind integrins (with its FERM-domain head) and actin (with an actin-binding site at the C-terminus of its long rod domain; Critchley, 2009). Talin feeds back to promote integrin activation and is required for the recruitment of numerous cytoplasmic adhesion proteins (Zhang et al., 2008; Zervas et al., 2011). Of particular interest is the force-dependent recruitment of vinculin. *In vitro* work has established that stretching the rod of talin exposes previously hidden vinculin-binding sites (VBSs, single helices within the α-helical bundles that make up the rod; Bass et al., 1999) that can then bind vinculin (Papagrigoriou et al., 2004; del Rio et al., 2009). Consistent with this model, the recruitment of vinculin to adhesions in cell culture is particularly sensitive to myosin II inhibition (Riveline et al., 2001; Pasapera et al., 2010; Carisey et al., 2013).

A series of four-helical bundles (seven in vertebrates, six in invertebrates) make up the head domain of vinculin, which is linked by a partially disordered proline-rich region to the five-helical bundle of the tail (Bakolitsa et al., 2004; Borgon et al., 2004). Interaction sites for vinculin ligands have been mapped across the protein (reviewed in Ziegler et al., 2006). A key ligand is talin (Burridge and Mangeat, 1984), and the interaction has been narrowed to the first two four-helical bundles of the head, the D1 domain (also known as Vh1; Bois et al., 2006): the VBSs in talin bind to the first four-helical bundle of the D1 domain, transforming it into a five-helical bundle (Izard et al., 2004). This first bundle of D1 retains most of the VBS-binding activity of the D1 domain in a two-hybrid assay (Bass et al., 2002), suggesting it is the minimal talin-binding site, but the second bundle is also capable of binding some ligands (Nhieu and Izard, 2007), and the entire D1 domain is generally used as a minimal head domain (Humphries et al., 2007; Carisey et al., 2013). Vinculin is notable among integrin-associated proteins for also localising to cell–cell adhesions (Geiger et al., 1980), and this is mediated through an interaction of the head with either α- or β-catenin (Hazan et al., 1997; Watabe-Uchida et al., 1998). The flexible neck of vinculin binds proteins of the CAP and vinexin family (Kioka et al., 1999; Bharadwaj et al., 2013), among other ligands, and the tail binds to actin (Jockusch and Isenberg, 1981; Johnson and Craig, 1995b), the scaffolding protein paxillin (Turner et al., 1990; Wood et al., 1994), and the membrane lipid phosphatidylinositol 4,5-bisphosphate (PIP_2_) (Johnson and Craig, 1995a; Gilmore and Burridge, 1996), and also promotes dimerisation (Johnson and Craig, 2000; Janssen et al., 2006; Chinthalapudi et al., 2014). By simultaneously binding talin and actin, vinculin provides an additional link to the cytoskeleton, giving extra mechanical support to the adhesion. A strengthening role is consistent with the relatively milder effects of losing vinculin compared to losing talin in cells in culture (Xu et al., 1998b; Zhang et al., 2008) and in developing animals (Alatortsev et al., 1997; Xu et al., 1998a; Monkley et al., 2000; Brown et al., 2002; Bharadwaj et al., 2013).

Although vinculin has many binding partners, the full-length protein has little binding activity due to a head–tail interaction stabilising the inactive conformation (Johnson and Craig, 1994; Bakolitsa et al., 2004; Borgon et al., 2004; Cohen et al., 2005). Constructs that relieve this head–tail autoinhibition are hyperactive, and dramatically increase the size and stability of focal adhesions associated with activated integrins (Cohen et al., 2005; Humphries et al., 2007), as well as making the recruitment of adhesion proteins no longer sensitive to myosin II inhibition (Carisey et al., 2013). The talin-binding D1 domain alone is sufficient to produce these effects (Cohen et al., 2006; Humphries et al., 2007), and, reciprocally, reducing the ability of the D1 domain to bind talin eliminates them (Cohen et al., 2006; Humphries et al., 2007; Carisey et al., 2013). The vinculin tail adds additional activity: it is required for hyperactive vinculin to produce traction forces (Dumbauld et al., 2013) and reorient adhesions in response to polarised forces (Carisey et al., 2013). A key aspect of vinculin function is therefore its activation status, and its effects on cell behaviour might be caused by its action on talin as well as direct or indirect recruitment of proteins to adhesions. Although the impact of hyperactive vinculin on cellular behaviour has been well documented, the impacts of these changes on cells within the organism have yet to be addressed. A mutant that produces hyperactive vinculin in mouse has a milder version of the defects caused by absence of vinculin (Marg et al., 2010), but vinculin levels are also strongly reduced, making it difficult to separate loss- and gain-of-function effects.

To probe further how vinculin contributes to adhesion, we have used *Drosophila* to compare loss- and gain-of-function effects during development. We find that vinculin hyperactivity is far more deleterious to the organism than inactivity, and discover a new function for vinculin in bringing adhesion proteins together independently of the usual integrin cue. The D1–talin-rod interaction is crucial for the formation of these cytoplasmic adhesion subcomplexes, supporting a model where hyperactive vinculin ectopically activates talin in the cytoplasm by mimicking the effect of force on talin. Finally, we dissect the negative effects of hyperactive vinculin into two discrete activities: morphogenetic defects caused by its head domain, and lethality caused by its D1 domain.

## RESULTS

### Although absence of vinculin has minor consequences, hyperactive vinculin causes lethality through its D1 domain

**Fig. 1.**
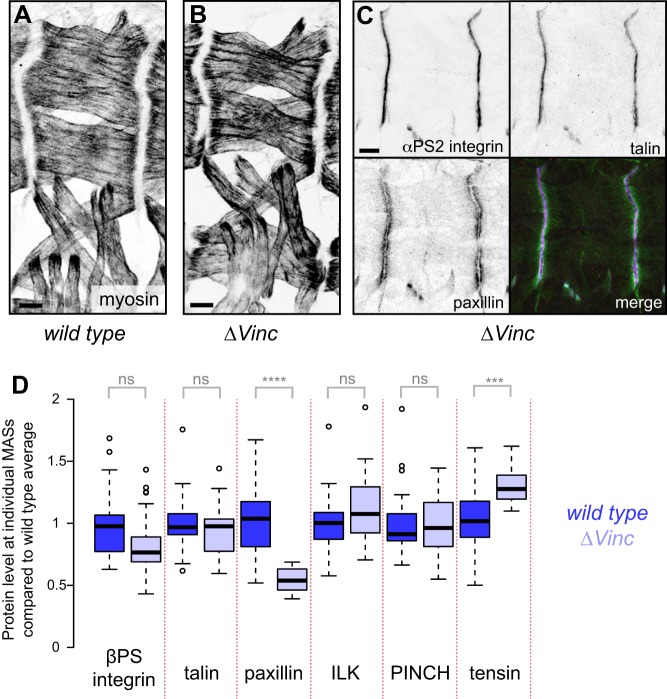
**Loss of vinculin has minimal effects on embryonic muscles.** Muscle morphology of late stage 16 wild-type (A) and Δ*Vinc* (B) embryos, stained for muscle myosin. (C) Recruitment of integrin (staining for the αPS2 subunit), talin–mCherry and paxillin–GFP to Δ*Vinc* MASs. Scale bars: 10 µm. (D) GFP- or YFP-tagged protein average fluorescence intensities for individual MASs compared to the wild-type average, using live stage 17 embryos. The box represents the 25–75th percentiles, and the median is indicated. The whiskers extend to data points that are less than 1.5× the interquartile range (IQR) away from 1st and 3rd quartile. Circles represent outliers. *n*=28 MAS measurements in ≥5 embryos per genotype. Dark blue, wild-type MASs, showing extent of variation; pale blue, Δ*Vinc* MASs. ***P≤0.001; ****P≤0.0001; ns, not significant (Student's *t*-test).

Flies homozygous for an inversion that disrupts the *Vinculin* (*Vinc*) locus are viable and fertile (Alatortsev et al., 1997), and we recently reported that a deletion removing the coding sequence, Δ*Vinc*, is also homozygous viable and fertile (Klapholz et al., 2015). Δ*Vinc* flies had normal embryonic muscles (Fig. 1A,B), without the detachments that typically arise from loss of integrin function. To examine whether there are any changes to the adhesion site in the absence of vinculin, we examined adhesion component levels. The αPS2βPS integrin (αPS2 is also known as Inflated and βPS as Myospheroid), talin and paxillin were still recruited to the muscle attachment sites (MASs; Fig. 1C). Levels of recruitment were then quantified using rescue constructs tagged with fluorescent proteins in live embryos. Levels of the integrin βPS subunit, talin, ILK and PINCH (also known as Steamer duck in flies) were not changed in Δ*Vinc* animals, whereas the levels of paxillin was reduced to about half, and those of tensin (also known as Blistery) were increased (Fig. 1D). Thus, in addition to paxillin contributing to vinculin recruitment (Pasapera et al., 2010), the converse is also found, and the elevation of tensin suggests that tensin and vinculin compete for limiting recruitment sites. As previously reported (Bharadwaj et al., 2013), CAP (the single fly orthologue of the CAP and vinexin family) is lost from MASs in Δ*Vinc* larvae, but we found it was still recruited normally in the embryo (Fig. S1), demonstrating a stage specificity of this interaction. Thus, although there are some alterations in adhesion protein levels in the absence of vinculin, embryonic muscle development proceeds normally.

To test whether the muscles functioned normally in fully grown larvae (third larval instar) lacking vinculin, we used time-lapse imaging to measure their crawling, but did not detect any impairment in crawling velocity (Fig. S1E). We then examined adult tissues; wings appeared normal (data not shown), but the indirect flight muscles showed a defect in the distribution of the actin at the muscle termini. In these muscles, actin accumulates at the MAS, but in Δ*Vinc* mutants the actin was frayed and expanded (Fig. S1F). The exceptional sensitivity of this tissue to the absence of vinculin might reflect its exceptional biomechanical activity during flight. This defect shares some similarity with the mild muscle phenotype in larvae reported by Bharadwaj et al. (2013). Thus, *Drosophila* vinculin does have functional roles, albeit minor ones under laboratory conditions.

**Fig. 2.**
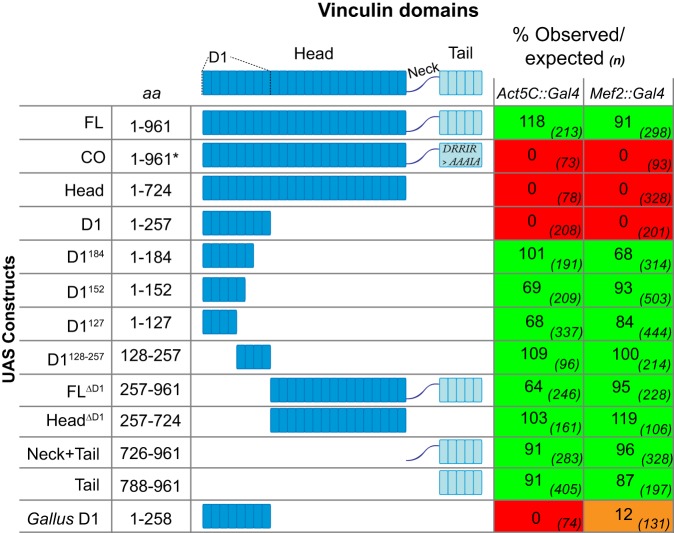
**Expression of hyperactive vinculin causes lethality through its D1 domain.** Vinculin domains are shown, with each block representing a single α-helix. The table shows details of regions of vinculin expressed through a UAS promoter, with amino acid numbers. Viability is shown as percentage observed compared to expected, calculated by comparing the number of *Gal4>UAS* flies to their balancer siblings. The number of flies counted is given in parentheses.

We then addressed the consequences of too much vinculin activity by using the UAS-Gal4 system (Brand and Perrimon, 1993) to express vinculin constructs with differing capacity for autoinhibition, each C-terminally tagged with TagRFP. Expression of wild-type, full-length vinculin (UAS vinc-FL) either in the muscles (with *Mef2::Gal4*) or ubiquitously (with *Act5C::Gal4*) did not affect viability (Fig. 2; calculated as relative eclosion of adults compared to heterozygous siblings). In contrast, expression of a constitutively open form (vinc-CO), where autoinhibition has been reduced by mutation of the ‘T12’ cluster of charged residues in the tail to alanine residues (Cohen et al., 2005), was lethal with either driver (no adults eclosed; Fig. 2). Thus, within the whole animal, too much vinculin activity is more deleterious than either its absence or an excess of the wild-type protein.

To address which portions of vinc-CO mediated lethality, we designed a set of constructs to express the different domains. Like vinc-CO, expression of the head was lethal (Fig. 2). In contrast, expression of the tail, alone or when connected to the flexible neck region, had no effect on viability (Fig. 2). Thus, as assayed by induction of lethality, head–tail autoinhibition is required to limit the activity of the head. As vinc-CO and vinc-Head behave identically in this assay, and in others described below, the ‘T12’ change appears equivalent to deleting the tail.

Within head of vinculin, the D1 domain contains all known binding sites for integrin-associated ligands. Expression of a construct encompassing this domain (UAS vinc-D1) was also lethal (Fig. 2), whereas expression of constructs lacking the D1 domain (UAS vinc-Head^ΔD1^ and UAS vinc-FL^ΔD1^), were not lethal (Fig. 2). Thus, the lethality of the head domain maps solely to the D1 domain.

Although the eight-helical D1 domain is generally used as the minimal functional domain of the head, VBSs bind the first four-helical bundle (Izard et al., 2004), and D1 truncations retain VBS-binding activity (Bass et al., 2002). We thus tested the activity of smaller constructs, reducing the eight helices of D1 to six (amino acids 1–184, UAS vinc-D1^184^), five (amino acids 1–152, UAS vinc-D1^152^) and four (amino acids 1–127, UAS vinc-D1^127^). The second bundle of D1 also has ligand-binding activity (Nhieu and Izard, 2007), so we also made a construct with this bundle on its own (amino acids 128–257, UAS vinc-D1^128-257^). However, when expressed with *Mef2::Gal4* or *Act5C::Gal4*, none of these D1 truncations caused lethality (Fig. 2), and hence the whole D1 domain is the minimal lethal domain.

Finally, we tested whether the lethal effect of hyperactive vinculin was conserved by expressing the D1 domain from chicken (*Gallus* vinc-D1; only five residues different from human vinc-D1) in flies. *Gallus* vinc-D1 was completely lethal with *Act5C::Gal4* and almost entirely lethal with *Mef2::Gal4* (Fig. 2), consistent with the high level of conservation of the D1 domain between invertebrates and vertebrates (55% identical). In summary, the D1 domain mediates the lethality of hyperactive vinculin *in vivo*.

### Hyperactive vinculin forms cytoplasmic aggregates that are adhesion subcomplexes

**Fig. 3.**
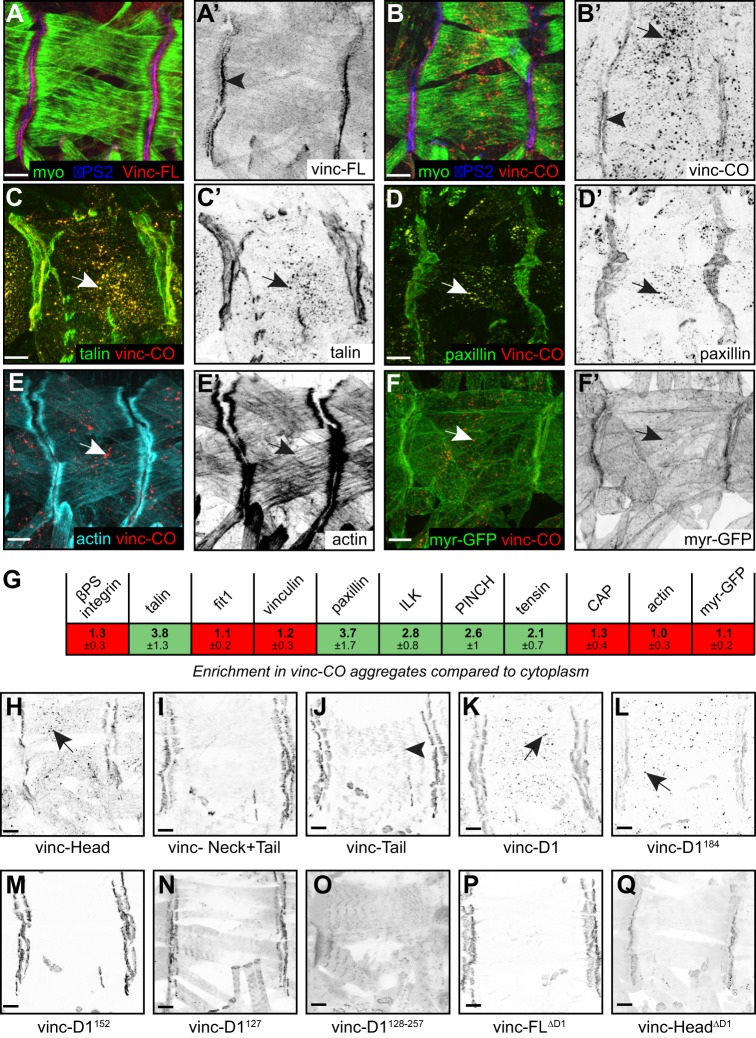
**Distribution of vinculin constructs in muscles.** Vinculin constructs were expressed using *Mef2::Gal4* in embryonic late stage 16/early stage 17 muscles (A–F), or newly hatched L1 larvae (H–Q). (A,A′) Vinc-FL, stained for muscle myosin and αPS2 integrin; the arrowhead shows MAS enrichment. (B,B′) Vinc-CO, stained for muscle myosin (myo) and αPS2 integrin; the arrowhead shows MAS enrichment, the arrow shows cytoplasmic aggregates. (C,C′) Vinc-CO expressed with GFP–talin; arrows show enrichment of talin in aggregates. (D,D′) As in C, with paxillin–GFP. (E,E′) Vinc-CO-expressing embryos stained with phalloidin to detect filamentous actin; the arrows highlight that there is no actin enrichment in the aggregates. (F,F′) Vinc-CO coexpressed with myristolated (myr-)GFP; the arrows highlight that there is no myr-GFP enrichment in the aggregates. (G) Enrichment of adhesion components in the aggregates calculated by comparing GFP signal in individual aggregates to the average signal in cytoplasm, and taking an average of these ratios, with the standard deviation shown below. *n*=40 aggregates measured per embryo, 4–5 embryos per genotype. (H–Q). Single channel images of vinculin constructs expressed in the muscles; arrows indicate cytoplasmic aggregates. The arrowhead in J shows association of vinc-Tail with sarcomeric actin. Scale bars: 10 µm.

To discover why vinculin hyperactivity was deleterious, we examined the distribution of the proteins *in vivo*. In late stage 16/early stage 17 embryos (15–18 h after egg laying), vinc-FL was recruited to MASs in muscles when expressed with *Mef2::Gal4* (Fig. 3A, arrowhead in Fig. 3A′) and showed a low, uniform level in the cytoplasm. This was similar to the localisation of wild-type vinculin expressed under its own promoter (Fig. S2A–A″), although Gal4-driven vinc-FL had a higher cytoplasmic level. In contrast, Gal4-driven vinc-CO formed aggregates throughout the cytoplasm of the muscle (Fig. 3B, arrow in Fig. 3B′), in addition to being enriched at the MAS (arrowhead in Fig. 3B′). These distributions were not tissue-specific: wild-type vinculin was recruited to adhesions in epithelial cells of the pupal wing and follicular epithelium of the ovary (Fig. S2B–E″), whereas vinc-CO additionally formed cytoplasmic aggregates (Fig. S2F–G′).

To investigate the nature of the cytoplasmic aggregates, we first examined whether they were caused by protein misfolding and degradation, but they did not colocalise with markers of lysosomes (LAMP1 in Fig. S3A,A′), autophagy (HuLC3, Fig. S3B,B′), or early, late or recycling endosomes (Fig. S3C–E′). Aggregation was not caused by TagRFP, as other TagRFP-tagged constructs did not form aggregates (for example, vinc-FL in Fig. 3A, and other constructs below), and aggregates were also formed by vinc-CO tagged with GFP (Fig. S3F). TagRFP is modified to be monomeric (Merzlyak et al., 2007).

We next tested the effects of vinc-CO on the distribution of adhesion proteins, by examining the products of GFP-tagged genomic rescue constructs. We started with the well-established vinculin ligands talin (Fig. 3C) and paxillin (Fig. 3D). In the presence of vinc-CO, these proteins showed a dual localisation: at their usual site of function at MASs, and ectopically in the cytoplasmic aggregates (Fig. 3C′,D′; compare to Fig. 1C) at a level >3× background levels in the cytoplasm (Fig. 3G). Among other adhesion proteins, ILK, PINCH and tensin were recruited to the aggregates, whereas endogenous vinculin, CAP and fermitin1 (fit1, an orthologue of kindlin) were not (Fig. 3G). A key function of cytoplasmic adhesion complexes is to link integrins to actin, but the aggregates formed by vinc-CO contained neither integrins (antibody staining for αPS2 integrin in Fig. 3B, βPS-integrin–GFP in Fig. 3G) nor actin (as detected with phalloidin, Fig. 3E,E′,G). The aggregates were not clearly associated with membranes as detected with myristolated GFP (Fig. 3F,F′,G). Thus, vinc-CO-stimulated aggregates represent cytoplasmic adhesion subcomplexes that appear to be uncoupled from both the membrane and the cytoskeleton. This contrasts with experiments in mammalian cells, where vinc-CO expands focal adhesions at the membrane containing both integrin and actin (Cohen et al., 2005; Humphries et al., 2007). Among recruited components of the subcomplex are proteins not known to be vinculin ligands (for example, ILK), suggesting a combination of direct and indirect recruitment events.

To define the minimal vinculin fragment capable of forming aggregates, we examined the distribution of truncated vinculins (see diagrams in Fig. 2) in newly hatched L1 larvae. Like vinc-CO, vinc-Head formed aggregates (arrow in Fig. 3H). In contrast, tail constructs did not (Fig. 3I,J), ruling out a contribution of tail–tail dimerisation to aggregation. The D1 domain also formed aggregates (Fig. 3K). Of the non-lethal constructs, only vinc-D1^184^ formed cytoplasmic aggregates (Fig. 3L–Q). However, vinc-D1^184^ aggregates did not recruit talin or paxillin (Fig. S3G–H′), suggesting that they are distinct from those formed by other constructs, nor did they colocalise with lysosomes (Fig. S3I,I′). As vinc-D1^184^ contains only half of the second four-helical bundle domain in D1, the exposed hydrophobic surface might promote aggregation through homo-oligomerisation. The recruitment of D1 subfragments to the MAS indicates that they still bind adhesion proteins, and thus are likely to be folded properly. We thus define D1 as the minimal domain of vinculin capable of forming cytoplasmic aggregates of adhesion proteins, in addition to being the minimal lethal domain.

### Active vinculin bypasses integrins to form aggregates through the talin rod

**Fig. 4.**
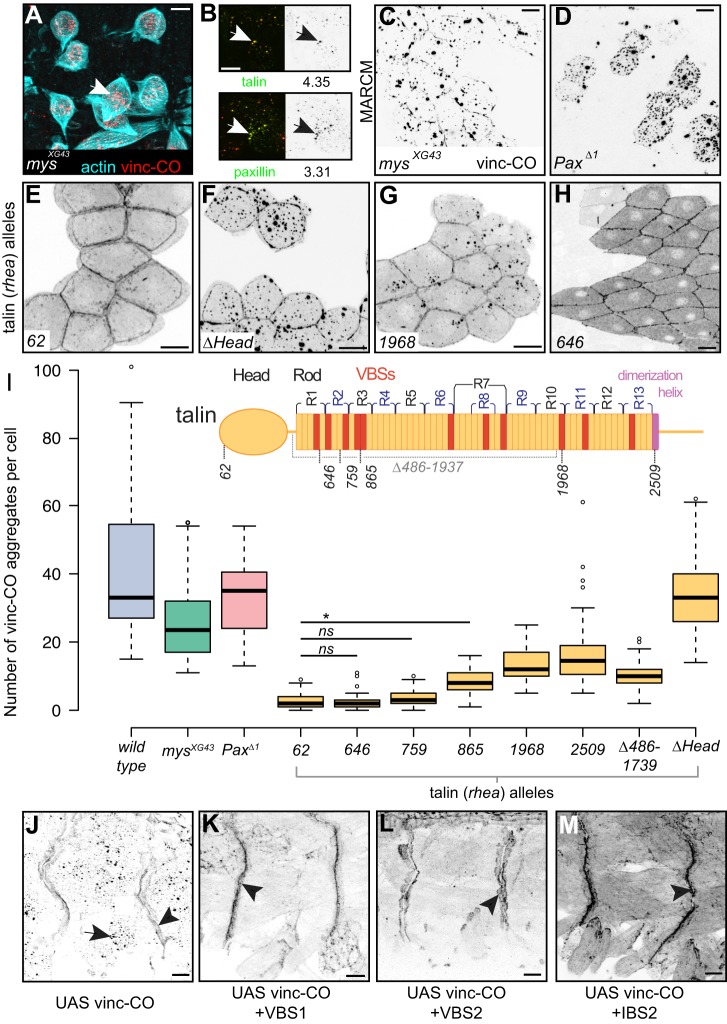
**The talin rod domain is required for aggregate formation.** (A) Integrin mutant (*mys^XG43^*) embryo expressing vinc-CO in the muscles, with actin detected by phalloidin. The arrow shows aggregates. (B) GFP–talin and paxillin–GFP in *mys^XG43^* embryos expressing vinc-CO; arrows show recruitment to aggregates; the average enrichment of protein in aggregates as compared to cytoplasm is given below the image. (C) MARCM clone in the follicular epithelium, mutant for βPS integrin and expressing vinc-CO, vinc-CO channel shown. (D) MARCM clone of cells mutant for paxillin and expressing vinc-CO. (E–H) MARCM clones expressing vinc-CO in talin mutant backgrounds indicated in the panel. (I) Number of aggregates per cell counted for MARCM clones mutant for integrin, paxillin or talin (various alleles). The inset shows talin domain structure with VBS helices in red, the dimerisation helix in pink, and position of the mutant truncations. Significance calculated as in Table S1, *P≤0.05; ns, not significant. Boxes, whiskers and circles are as described in Fig. 1. Number of cells counted per genotype: WT=39; *mys^XG43^*=54; *Pax*^Δ*1*^=23; talin alleles: *62*=70, *646*=65, *759*=53, *865*=42, *1968*=75, *2509*=64, Δ*486-1739=*50, Δ*Head*=38. Over four clones in at least four individual egg chambers per genotype. (J–M) Vinc-CO expressed on its own (J), with one copy of VBS1–GFP (K), two copies of VBS2–GFP (L), or one copy of GFP–IBS2 (M); the vinc-CO channel is shown. Arrows shows cytoplasmic aggregates; arrowheads show MAS localisation. Scale bars: 10 µm.

The formation of cell–matrix adhesions involves integrin recruitment of cytoplasmic adhesion proteins. Although the vinc-CO aggregates did not contain integrins, integrins might still play a role in seeding the complexes. We tested this by genetically removing integrins, and found no effect on aggregate formation: in integrin mutant embryos (*mys^XG43^*, a null allele of the gene encoding the βPS integrin subunit; orthologous to the β1 integrin subunit), vinc-CO still formed aggregates in the detached and rounded up muscles (Fig. 4A). Furthermore, vinc-CO was still capable of recruiting talin and paxillin in the absence of integrins (Fig. 4B). Thus, surprisingly, vinc-CO can induce adhesion subcomplexes without integrins. This suggests that vinculin is providing an adhesion complex trigger that functions in parallel with integrins, and, since vinculin is not needed to make adhesions, it must be acting redundantly with integrins.

To further define the molecular requirements for aggregate formation, we examined a different cell type, the follicle cells of the ovary, so that we could use the MARCM technique (Lee and Luo, 2001) to generate clones of cells which lack a given adhesion protein and express vinc-CO. Vinc-CO formed aggregates in follicle cells when expressed in wild-type cells (Fig. S2G). As we found in the embryo, vinc-CO formed aggregates in the absence of integrins (Fig. 4C), although the number of aggregates per cell was decreased (Fig. 4I; statistical comparison of aggregate-forming capacity in Table S1; note that the aggregate size was too variable to be a useful measure and a change in aggregate number did not correlate with change in cell size). Loss of paxillin also had little effect on vinc-CO aggregates (Fig. 4D,I). In contrast, removal of talin (null talin^62^ truncation; Klapholz et al., 2015) led to an almost complete loss of aggregates, with vinc-CO being more uniform in the cytoplasm, and enriched at cell–cell junctions (Fig. 4E,I). Aggregates of vinc-Head and vinc-D1 were also much reduced in the absence of talin (Fig. S4A,B), suggesting that a D1–talin interaction is the initial event in generating aggregates. We confirmed that talin was required for aggregate formation in embryos (Fig. S4C). Talin is therefore required for hyperactive vinculin to generate cytoplasmic aggregates. The increased association of vinc-CO with cell–cell junctions in the absence of talin is consistent with an interaction with α- or β-catenin, but the aggregate pathway appears to be mediated exclusively through talin.

We then mapped the regions of talin required for vinc-CO to induce aggregates. The head domain of talin interacts with integrins and the membrane, and its rod domain is divided into R1–R13 domains containing 11 VBSs and a C-terminal dimerisation helix (diagram in Fig. 4I; Critchley, 2009). We utilised a series of talin deletion mutations (Klapholz et al., 2015), and assayed the capacity of vinc-CO to make aggregates in clones of cells that express only the truncated talin. Removing the head did not eliminate aggregates (Fig. 4F,I), showing that the rod alone is a sufficient platform for aggregate formation. We next investigated how much of the rod domain was required. The smallest deletion, talin^2509^, retains all of its VBSs but has the dimerisation domain partly deleted (Klapholz et al., 2015). This caused a reduction in the number of aggregates formed by vinc-CO (Fig. 4I; Fig. S4C), presumably caused by the reduction of 22 to 11 VBSs following loss of dimerisation. Decreasing the rod length with progressively larger deletions resulted in a correlated decrease in the number of aggregates per cell (Fig. 4G,I). However, C-terminal deletions containing only the first two VBSs, talin^759^, or just the first, talin^646^, had the same effect as the null: an almost complete loss of aggregates (Fig. 4H,I). Thus, a talin with one or two VBSs in the rod is not a sufficient platform for aggregates. The next truncation, talin^865^, which has four VBSs, supports aggregate formation (Fig. 4I), albeit to a low level. To test whether the N-terminal region of the rod specifically is necessary for aggregation, we generated a complementary construct, talin^Δ486-1937^, which retains three C-terminal VBSs and the dimerisation helix. This supported aggregate formation to the same extent as talin^865^ (Fig. 4I), showing that no single region of the rod is required. Thus, the rod domain of talin is required for aggregate formation, and multiple sites along the rod appear to contribute to the process.

Considering talin was required for vinc-CO to make aggregates, we investigated whether this required interaction between talin and the talin-binding site in vinculin. To outcompete binding to endogenous talin, we coexpressed VBSs, α-helices from the rod domain of talin that bind to the head of vinculin with high affinity (Bois et al., 2006). We designed GFP- or mCherry-tagged UAS constructs for VBS1 and VBS2 (helices 4 and 12, respectively; Gingras et al., 2005), and used a previously generated construct encoding four helices of the R11 bundle (helices 47–50) that contain a VBS (helix 50), known as GFP–IBS2 (Ellis et al., 2011). We observed an almost complete suppression of aggregates when vinc-CO was coexpressed with VBSs in embryonic muscles (Fig. 4J–M). This result confirms the talin^646^ result (Fig. 4G) in demonstrating that a single VBS is not a sufficient platform for vinc-CO to make aggregates. We found a similar loss of aggregates when vinc-CO–GFP was coexpressed with mCherry-tagged VBS1 (Fig. S4D,E). Finally, vinc-Head and vinc-D1 aggregates were also lost upon VBS coexpression (Fig. S4F–I).

Taken together, these results suggest that VBS binding is required for vinc-CO to make aggregates. Although there are other proteins that contain VBSs, considering the loss of aggregates upon talin removal, we favour talin as the key binding partner.

### Aggregate formation corresponds with ability of vinculin to bind cytoplasmic talin

**Fig. 5.**
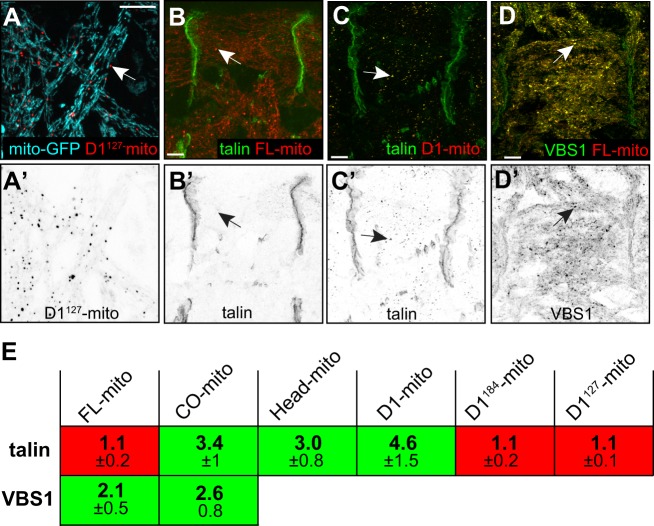
**Hyperactive vinculin can recruit talin to an ectopic location.** (A,A′) Coexpression of GFP with a mitochondrial import signal (mito-GFP) and vinc-D1^127^-RFP-mito; the arrow shows vinc-D1^127^-RFP-mito aggregates on the mitochondrial surface. (B,B′) Expression of vinc-FL-RFP-mito in a GFP–talin background; the arrow shows failure of talin recruitment. (C,C′) Expression of vinc-D1-RFP-mito in a GFP–talin background; the arrow shows talin recruitment. (D,D′) Coexpression of vinc-FL-RFP-mito with GFP–VBS1; the arrow shows GFP–VBS1 recruitment. (E) Enrichment of GFP–talin or GFP–VBS1 in mitochondrial aggregates compared to background cytoplasm, calculated as in Fig. 3, with standard deviation below. *n*=40 measurements per embryo, in 4–5 embryos per genotype. Scale bars: 10 µm.

Our results suggest that vinc-CO binds to talin rod through its D1 domain to form aggregates. Neither full-length vinculin nor D1 truncations formed talin-containing aggregates, suggesting that they cannot bind talin in the cytoplasm. To investigate this further, we used an alternative approach to assess talin binding, targeting vinculin proteins to the mitochondria with the C-terminal outer membrane anchor from *Listeria monocytogenes* ActA (Pistor et al., 1994; Bubeck et al., 1997; Cohen et al., 2006), and testing whether they could recruit talin. Most targeted vinculins formed discrete aggregates on the mitochondria (Fig. 5A), whether or not the non-targeted forms formed cytoplasmic aggregates, except for vinc-D1^152^-mito, which therefore was not used. While the lethal, aggregate-forming vinculins recruited talin to the mitochondria (Fig. 5B,B′,E), vinc-FL, vinc-D1^184^ and vinc-D1^127^ did not (Fig. 5C,C′,E). Thus, the ability to form cytoplasmic aggregates with talin correlates with recruitment of cytoplasmic talin to the mitochondria.

To test whether force-dependent opening of talin was sufficient for it to bind to closed vinculin, we examined whether an isolated VBS, mimicking open talin, could bind to closed vinculin. GFP–VBS1 was strongly recruited to vinc-FL-mito (Fig. 5D,E), and, as expected, to open vinc-CO-mito (Fig. 5E). Thus, in this system, binding between vinculin and talin requires only one to be activated, either by exposure of a talin VBS or loss of vinculin autoinhibition.

### Developmental defects of hyperactive vinculin – decoupling aggregates from lethality

**Fig. 6.**
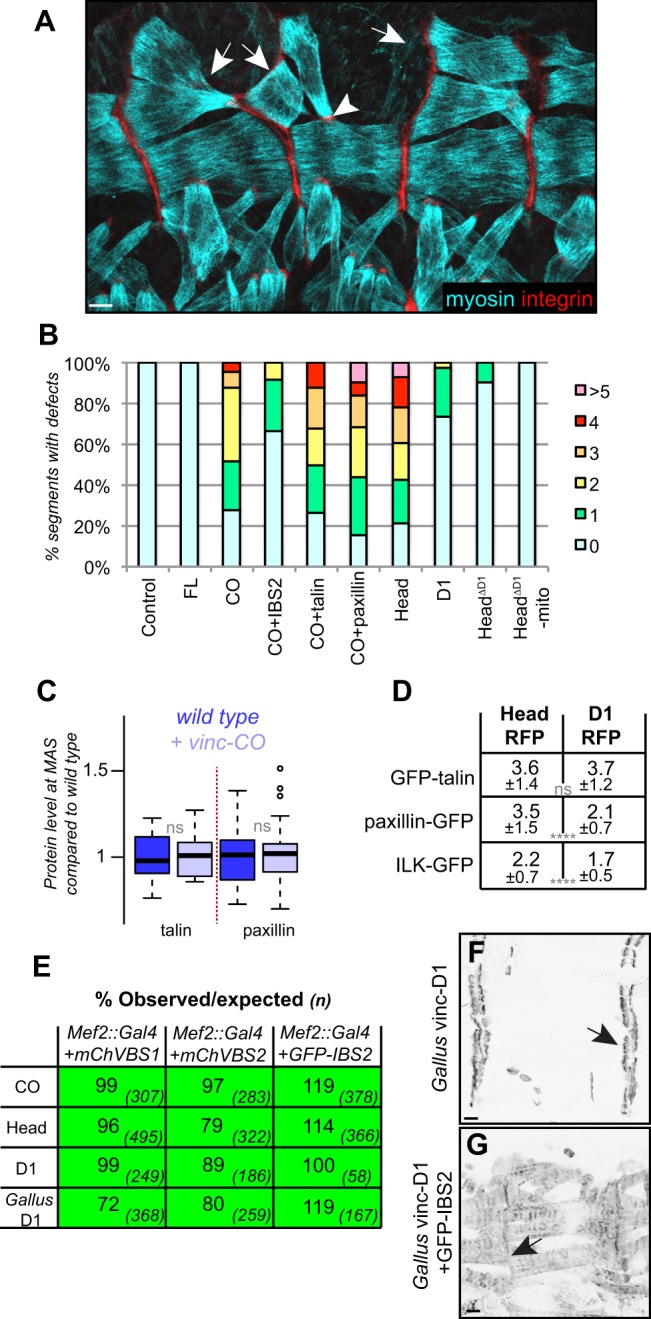
**The effects of hyperactive vinculin on muscle development and viability.** (A) Defects in muscle morphology following vinc-CO expression, as revealed by myosin and integrin staining; arrows show changes in muscle pattern, the arrowhead shows ectopic attachment. (B) Quantification of muscle defects resulting from expressing different vinculin constructs, shown as percentage of segments with x number of defects as colour coded. Number of segments counted: control=23, FL=40, CO=25, CO+IBS2=24, CO+talin=34, CO+paxillin=32, Head=28, D1=42, Head^ΔD1^=32, Head^ΔD1^-mito=34. Stage 16 embryos, ≥5 embryos per genotype. (C) Effect of vinc-CO expression on levels of talin and paxillin at the MAS, shown as average fluorescence intensities for individual MASs compared to the wild-type average. Boxes, whiskers and circles are as described in Fig. 1. Dark blue, wild-type individual MASs, to show variation; pale blue, levels in the presence of vinc-CO. *n*=34 (talin) and 26 (paxillin) measurements in four newly hatched L1 larvae. ns, not significant (Student's *t*-test). (D) Level of recruitment of talin, paxillin and ILK to vinc-Head versus vinc-D1 cytoplasmic aggregates, calculated as in Fig. 3, with standard deviation below. *n*=40 measurements per embryo, in 4–5 embryos per genotype. ****P≤0.0001 (Student's *t*-test). (E) Rescue of lethality by coexpression of VBSs with lethal vinculin proteins. Viability is shown as percentage observed compared to expected, calculated by comparing *Gal4>UAS* flies to their balancer siblings. The number of flies counted is given in parentheses. (F) Newly hatched L1 larvae expressing *Gallus* vinc-D1 in the muscles. The arrow shows MAS localisation. (G) Newly hatched L1 larvae coexpressing *Gallus* vinc-D1 with GFP–IBS2 in the muscles; the arrow highlights the reduction in MAS enrichment. Scale bars: 10 µm.

Our data suggest that hyperactive vinculin generates aggregates through the rod of talin and that a vinculin–talin platform recruits other cytoplasmic proteins. As hyperactive vinculin is also recruited to adhesions (Fig. 3B), it might exert its negative effects on development in either of these locations or both. We examined the relative contribution of the two pools, first looking at the effects on muscle development. Expression of vinc-CO with *Mef2::Gal4* caused a range of phenotypes, including misshapen muscles, misplaced muscles, gaps in the muscle pattern and ectopic attachments (Fig. 6A, quantified in Fig. 6B; compare to wild-type muscles in Fig. 1A), whereas expression of vinc-FL did not cause these defects (Fig. 6B). Phenotypes were suppressed when VBSs were coexpressed (Fig. 6B), indicating that binding to talin, or another VBS-containing protein, mediates the effect.

We wondered whether sequestration of proteins by vinc-CO aggregates caused the muscle phenotypes. To test whether it is sequestration of talin and paxillin, we coexpressed them along with hyperactive vinculin, but this did not suppress the muscle phenotypes (Fig. 6B) nor alter aggregate formation (Fig. S4J,K). Furthermore, levels of talin and paxillin at MASs were not reduced when vinc-CO was expressed (Fig. 6C; Fig. S4L–M). Thus, sequestration of these adhesion proteins at least does not explain the muscle phenotypes, consistent with the defects not resembling typical integrin loss-of-function phenotypes such as muscle detachment (see Fig. 4A) or actin detachment from the membrane (Zervas et al., 2001). Either the aggregates sequester proteins involved in other muscle developmental processes, or vinc-CO directly impairs these processes, in aggregates or at the adhesion.

As VBSs suppressed both muscle phenotypes and aggregates, we expected the minimal aggregate-forming domain, D1, to induce muscle phenotypes. However, although vinc-Head caused equivalent muscle defects to vinc-CO, vinc-D1 had only minor effects (Fig. 6B). Thus, the D1 domain does not account for all the activities of the head domain *in vivo*. Given the difference between vinc-Head and vinc-D1, we analysed whether the rest of the head perturbed muscle development, but neither UAS-vinc-Head^ΔD1^-RFP (localised to MASs) nor UAS-vinc-Head^ΔD1^-RFP-mito (in aggregates on mitochondria) caused muscle defects (Fig. 6B). The whole head is thus the minimal domain capable of causing muscle phenotypes. Importantly, these data show that aggregates alone do not correlate with muscle defects. However, vinc-Head aggregates could be different to those produced by vinc-D1. We therefore examined recruitment of talin, paxillin and ILK, and found that the levels of paxillin and ILK were higher in the vinc-Head aggregates compared to the vinc-D1 aggregates (Fig. 6D). As the full head is more effective at sequestration, differences in recruitment capacity to the aggregates might explain its muscle phenotypes. Alternatively, it could have additional activities elsewhere in the cell, such as at the adhesion sites.

Finally, we investigated whether lethality of hyperactive vinculin required aggregate formation. Coexpression of VBSs suppressed the lethality of hyperactive vinculin (Fig. 6E), suggesting that interaction with talin (or another VBS-containing protein) mediates lethality. *Gallus* vinc-D1 lethality was also suppressed by *Drosophila* VBSs (Fig. 6E), indicating a common mechanism of lethality. VBS coexpression resulted in loss of *Gallus* vinc-D1 MAS localisation (Fig. 6F,G), suggesting it is recruited by binding a VBS. However, when expressed on its own, *Gallus* vinc-D1 did not form cytoplasmic aggregates (Fig. 6F). Thus, aggregates are not required to cause lethality. As all lethal constructs were localised at MASs, it seems likely that it is their action at the adhesion that causes lethality, but we cannot rule out activity elsewhere in the cell. Thus, we can distinguish two separate detrimental activities within vinc-CO: one causing muscle defects, which requires its whole head domain, and a second causing lethality, which is performed by its D1 domain.

## DISCUSSION

Whereas flies can tolerate the loss of vinculin, we have discovered that excessive vinculin activity is lethal, and causes defects in muscle development. Both of these deleterious effects appear to require binding a VBS-containing protein such as talin. Talin is also required for a new role of *Drosophila* vinculin: inducing the formation of cytoplasmic aggregates that are adhesion subcomplexes. These subcomplexes are not linked to integrins or the cytoskeleton, and demonstrate that adhesion protein complexes can form without any input from integrin.

Flies lacking vinculin displayed defects in the adult musculature, similar to the mild defects in larval musculature reported by Bharadwaj et al., (2013). Other tissues appeared normal, and our attempts to identify additional impairment in the athletic abilities of the flies were not successful, so the fly phenotype remains weaker than the phenotypes observed in mice, zebrafish or nematodes lacking vinculin (Barstead and Waterson, 1991; Xu et al., 1998a; Cheng et al., 2016). Redundancy with other adhesion proteins (such as talin; Klapholz et al., 2015) might explain the relatively mild phenotype of this highly conserved protein.

The contrast between the consequences of loss and gain of vinculin activity are striking. In general, overexpressed integrin-associated proteins do not induce lethality in *Drosophila* [for example, talin (Tanentzapf et al., 2006), tensin (Torgler et al., 2004), and ILK (Zervas et al., 2011)]. Two integrin-associated proteins cause lethality when the wild-type form is overexpressed: focal adhesion kinase (Grabbe et al., 2004) and parvin (Vakaloglou et al., 2012). The lethality of vinculin relied on a reduction of its autoinhibition, but this does not seem general to *Drosophila* adhesion proteins: disruption of talin autoinhibition has mild effects (Ellis et al., 2013), and whereas expression of tensin fragments does cause some phenotypes (Torgler et al., 2004), expression of fragments of other adhesion proteins does not (see above references). The severe effects of hyperactive vinculin fit with the very strong intermolecular interactions that keep it in the closed state. Activating mutations of vinculin have not, to the best of our knowledge, been reported in the human population, as expected if, as in flies, they cause dominant lethality.

Expressing vinc-CO or vinc-Head in the developing musculature led to developmental defects. These could arise as a result of hyperactive vinculin in the aggregates or at the adhesion site. The defects are distinct from integrin loss phenotypes, and this might reflect recruitment of additional proteins contributing to muscle formation to the aggregates or the adhesion. Cytoskeletal machinery is crucial for muscle fusion and muscle pathfinding to tendon cell targets (Maartens and Brown, 2015b), and an interaction between hyperactive vinculin complexes and more general cytoskeletal factors might explain the muscle defects. Sequestration of Z-disc proteins to ectopic intracellular aggregates has been implicated in the muscle phenotypes associated with myofibrillar myopathy (Ruparelia et al., 2016), and a similar effect may be stimulated by hyperactive vinculin.

Cytoplasmic aggregate formation appears to be unique to *Drosophila* vinculin. *Gallus* vinc-D1 did not generate cytoplasmic aggregates, even though it appeared to interact with *Drosophila* talin (coexpressing *Drosophila* VBSs blocked its recruitment and lethality). In vertebrate cell culture, hyperactive vinculin is recruited to integrin adhesions at the membrane (Cohen et al., 2005; Humphries et al., 2007; Carisey et al., 2013), but cytoplasmic talin-containing aggregates have not been reported. The interaction between vertebrate vinc-Head and talin rod *in vitro* requires prior stretching of the rod by force (del Rio et al., 2009; Ciobanasu et al., 2014; Yao et al., 2014), consistent with the *in vivo* interaction relying on prior talin stretching at the adhesion. In contrast, our mitochondrial targeting experiments indicate that activated *Drosophila* vinculin can bind to un-stretched talin in the cytoplasm. A prediction from these results is that vertebrate vinculin should not recruit talin to the mitochondria, whether active or not. However, in vertebrate cells, talin is recruited by mitochondrially targeted vinc-CO (Cohen et al., 2006) and even full-length vinculin (albeit very weakly; Bubeck et al., 1997). However, in these cases targeted vinculin constructs pull the mitochondria to the membrane, so that vinculin and talin become associated with integrins and actin (no such association was found in our targeting experiments). Thus, it seems feasible that, in these experiments, the association of vinc-CO is with stretched talin at the adhesion site, rather than with cytoplasmic talin as occurs in *Drosophila*.

Several lines of evidence show that hyperactive *Drosophila* vinculin formed aggregates by binding to cytoplasmic talin. In the absence of talin, no aggregates were formed, and the rod of talin was a sufficient platform for aggregation, with longer sections supporting more aggregates, presumably due to an increase in the number of VBSs available per talin molecule. VBS coexpression blocked aggregate formation, suggesting that direct binding between vinculin and talin was important, and indeed the minimal vinculin fragment capable of forming aggregates was the talin-binding D1 domain. Hyperactive, but not wild-type, vinculin was capable of recruiting talin to the mitochondrial surface. Vinc-CO recruitment of talin to the aggregates was not altered by the loss of integrins, ruling out an alternative hypothesis whereby an initial stretching of talin at the adhesion is a first step in the formation of the cytoplasmic aggregates.

**Fig. 7.**
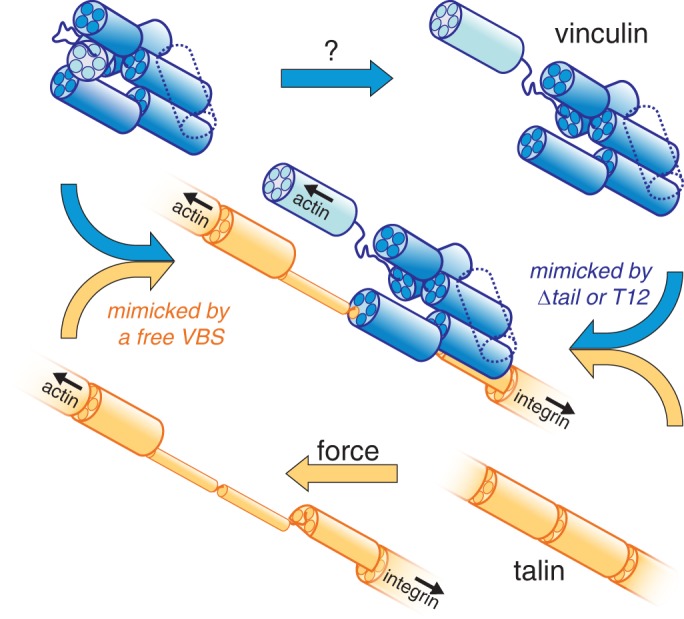
**Model for the interaction between vinculin and talin in *Drosophila*.** Interaction between vinculin and talin can be driven either by loss of vinculin autoinhibition, or by exposure of VBSs in the rod of talin. Bundles of talin rod in orange, and vinculin in blue, based on structures of human proteins (light blue bundle, vinculin tail domain; dotted bundle, single bundle present *Drosophila* vinculin instead of the two bundles in human vinculin).

An interesting feature of the vinculin–talin interaction is its reciprocity: just as hyperactive vinculin appears to bind closed talin, isolated VBSs can bind closed vinculin on the mitochondria, consistent with the capacity of vertebrate VBSs to dislodge the head from the tail *in vitro* (Bois et al., 2006). Thus, the interaction between vinculin and talin in *Drosophila* need only require activation of one partner (Fig. 7). An open question is whether there are normal signals, mimicked by the ‘T12’ mutation, that open *Drosophila* vinculin so that it can force talin into an extended conformation. Recently, Atherton et al., (2015) found that expression of a mutant talin with a deletion of domains R2–R3, which contain four VBSs, causes very similar effects to expressing vinc-CO. Binding of activated-vinculin thus alleviates some form of internal negative regulation within talin, which might in part be due to regulation of the central actin-binding domain encompassing R4–R8 (Atherton et al., 2015).

*Gallus* vinc-D1 demonstrated that hyperactive vinculin could induce lethality in *Drosophila* without forming aggregates. How it does so remains an open question, but we favour the idea that it is caused by the action of the D1 domain of vinculin on talin at the adhesion sites. The vinculin head stabilises talin into a stretched conformation in cells and *in vitro* (Margadant et al., 2011; Yao et al., 2014), and this relies on prior stretching of talin (Ciobanasu et al., 2014; Yao et al., 2014). Furthermore, vinculin is required for talin to extend fully away from the plasma membrane (Case, et al., 2015; Klapholz et al., 2015). Thus, lethality could arise from hyperactive vinculin binding to stretched talin and the failure of vinculin to release when force is reduced. Cycles of stretching and relaxation might be crucial for normal talin function or relaxation of talin might be required for its dynamic turnover. Alternatively, hyperactive vinculin might stimulate too much adhesion, stabilising integrin adhesions and reducing turnover in dynamic morphogenetic events. Elevated integrin expression has been shown to hinder cell migration in the *Drosophila* ovary (Lewellyn et al., 2013), and vinculin stimulation of integrin activation might affect similar processes. The lethality caused by vinc-D1 constructs occurs without defects in muscle morphogenesis. Assessing whether the muscle defects of vinc-Head and vinc-CO also contribute to lethality would require a method to block the lethality of vinc-D1 without impairing the muscle phenotypes of vinc-Head or vinc-CO, which we currently lack.

Although we have only examined vinculin D1 from two species, we speculate that vertebrate vinculin has lost the ability to bind to closed talin, and might have become more tightly closed by the addition of an eighth four-helix bundle that occurred during the evolution of the deuterostome lineage (our unpublished analysis). Thus, vertebrate cells might be even more sensitive to the consequences of aberrant association between vinculin and talin.

Our results suggest that certain proteins have the ability to act as a switch, triggering assembly of an integrin adhesion complex. Integrins are well known to have this switch ability: engagement with the ECM and clustering triggers the formation of adhesion sites (Miyamoto et al., 1995). When *Drosophila* vinculin loses autoinhibition, it triggers the assembly of an adhesion complex, and this process can occur entirely independently of integrins. In contrast to integrins, however, the full complement of adhesion proteins is not recruited, suggesting that additional mechanisms are required (for instance, membrane proximity, application of force, or signalling). This raises the question of how the additional proteins are recruited to the cytoplasmic aggregates, and whether the pathways involved are similar to those utilised by constitutively active vinculin at adhesions (Carisey et al., 2013) and by integrins and talin in normal adhesions. Recruitment requires talin, but the relative contributions of vinculin and talin have yet to be established.

Integrin-independent interactions of adhesion proteins have been demonstrated by fluorescence correlation analysis wherein adhesion components self-assembled in the cytosol (Hoffmann et al., 2014). However, these ‘building blocks’ were composed of three or four protein species, never assembled into larger structures and did not include a talin–vinculin interaction. Nevertheless, the above work shows how interactions between the component parts of the adhesion need not necessarily rely on a direct or even indirect link to integrins, consistent with our work. A key role of integrins might be to trigger the assembly of the cytoplasmic adhesion-complex-specific sites in the membrane, rather than being a necessary part of this link.

From an evolutionary perspective, certain cytoplasmic components like vinculin and talin predate the integrins (Kreitmeier et al., 1995; Sebe-Pedros et al., 2010). It is tempting to propose that integrins co-opted pre-existing cytoplasmic complexes, using them to strengthen their adhesion to the ECM at discrete sites along the cell surface. This evolutionary change may also have required mechanisms to restrict the spontaneous formation of adhesion-like complexes in the cytoplasm. The strong head–tail interaction of vinculin could be one such mechanism.

## MATERIALS AND METHODS

### Fly genetics

*Drosophila melanogaster* stocks used in this study: *Mef2::Gal4*, *Act5C::Gal4*, *UAS::GFP-Lamp1*, *UAS::eGFP-huLC3*, *UAS::myr-GFP*, *UAS::mito-GFP* (all sourced from the Bloomington *Drosophila* Stock Center, Indiana University); ΔVinc, *βPS-GFP*, *GFP-talin* (all Klapholz et al., 2015); *mys^XG43^* (Jannuzi et al., 2002); pax^Δ1^ (Bataille et al., 2010); *talin-mCherry* (Venken et al., 2011); *UAS::talin* (Tanentzapf et al., 2006); *UAS::GFP-IBS2* (Ellis et al., 2011); *paxillin-GFP* (Bataille et al., 2010); *UAS::paxillin* (Vakaloglou et al., 2012); *tensin-GFP* (Torgler et al., 2004); *ILK-YFP* (unpublished, made as *ILK-GFP* in Zervas et al., 2001); *PINCH-GFP* (Kadrmas et al., 2004); *Fermitin1-GFP* (a genomic fragment, −1579 to +3942 relative to the ATG, with mGFP6 inserted following residue 228 and with three-serine linkers on each side; a gift of Danelle Devenport, The Gurdon Institute, University of Cambridge, UK). To misexpress vinculin constructs, the UAS-Gal4 system was used (Brand and Perrimon, 1993). To remove maternal and zygotic contributions of talin and paxillin, the dominant female sterile technique was used (Chou and Perrimon, 1996). To generate follicle cell clones lacking βPS and expressing Vinc-CO-RFP, the MARCM system was used (Lee and Luo, 1999). *hsFlp*, *Tub-Gal80^ts^*, *FRT19A; Tub-Gal4*, *UAS cd8GFP/CyO* flies were crossed to *mys^XG43^*, *FRT19A; UAS Vinc-CO-RFP/CyO* flies, pupae were heat shocked for 2 h in a 37°C water bath, and non-CyO adult females selected and dissected. *rhea* and *pax* mutant clones expressing Vinc-CO were generated in the same manner with different MARCM lines for each FRT (FRT2A and FRT40A, respectively). To test the domains of talin required for vinc-CO aggregation, we employed a series of *rhea* deletions as well as a null allele rescued by construct lacking the head or having the internal deletion in the rod (Klapholz et al., 2015). For controls, wild-type FRT chromosomes were used in place of mutant chromosomes. To generate Flp-out clones (Struhl and Basler, 1993) expressing Vinc-CO or Vinc-Head in the follicle cells and wing, *hsFlp; arm::FRTstopf+FRTGal4* (details available upon request) flies were crossed to *UAS-vinc-X* flies and progeny heat shocked either as L1 larvae (for clones in the wing) or pupae (for clones in the follicle cells). Fluorescence from the vinculin constructs was used to mark clones.

### Larval crawling assay

Wandering third-instar larvae were placed on 2% agar plates and allowed to acclimatise for 10 s. 1 min movies were captured at 30 frames per second with a JVC TKC1380 camera mounted on a Leica M8 Dissecting microscope. A custom-made Fiji script (available from the corresponding author upon request) was used to track larvae and calculate average velocity.

### Generation of tagged vinculin and talin constructs

Generation of new *Vinc* alleles was achieved by imprecise P-element excision and is described in Klapholz et al. (2015) along with the construction of endogenous tagged vinculin constructs. To generate UAS controlled wild-type vinculin constructs (*UAS::vinc-FL*), the *Vinc* coding sequence was cloned into UASp with C-terminal GFP or TagRFP tags. To generate constitutively open vinculin (UAS vinc-CO), the ‘T12’ set of charge-to-alanine mutations (DRRIR>AAAIA, amino acids 869–873; as in Cohen et al., 2005) were introduced into the tail of genomic full-length vinculin and cloned into the UASp vector with a C-terminal GFP or TagRFP tag. Both *UAS::vinc-FL* and *UAS::vinc-CO* were integrated by untargeted P-element insertion, and insertions with good expression levels were selected for analysis*.* To generate vinculin truncation constructs, the relevant sequences were amplified by PCR from genomic DNA, and cloned into UASp AttP vector, and all inserted into the genome in the same position (landing site on chromsome 2R: 51D). The coordinates of these constructs in the genomic DNA (where 1=ATG) are: vinc-FL/CO, 1–3135; vinc-Head, 1–2172; vinc-D1, 1–771; vinc-D1^184^, 1–552; vinc-D1^152^, 1–456; vinc-D1^127^, 1–381; vinc-D1^128-257^, 381–771; vinc-Head^ΔD1^, 769–2172; vinc-FL^ΔD1^, 769–3135; vinc-Neck+Tail, 2176–3135; vinc-Tail, 2423–3135. *Gallus gallus* vinc-D1–RFP (amino acids 1–258) was generated similarly from a cDNA clone (Carisey et al., 2013). To generate mitochondrially targeted vinculin constructs, the C-terminal membrane anchor from the *Listeria* ActA protein (Pistor et al., 1994) linked to TagRFP with a polyserine linker was synthesised (Mr Gene), and cloned with vinculin fragments into the UASp AttP vector for insertion into the 51D landing site. To generate talin VBS constructs, we amplified sequences coding for VBS1 (helix 4 of the rod), VBS2 (helix 12 of the rod) by PCR, and cloned them into the UASp vector with an N-terminal GFP or mCherry tag. The GFP–talin^Δ486-1739^ genomic rescue construct was made by the same strategy as for the wild-type GFP-tagged construct described in Klapholz et al. (2015).

### Immunohistochemistry and imaging

Embryos, ovaries, wings and adult flight muscles were staged, fixed, stained and imaged using standard protocols, with sodium deoxicholate added to the wash for the Rab protein stainings as in Gomez-Lamarca et al. (2015). Antibodies used were: paxillin (rabbit, 1:200; Chen et al., 2005); αPS2 integrin (rat, 1:10; Bogaert et al., 1987); muscle myosin II (mouse, 1:100; Kiehart et al., 1990); E-cadherin (rat, 1.100; Developmental Studies Hybridoma Bank, University of Iowa); CAP (rabbit, 1.1000; Bharadwaj et al., 2013); Rab5 (rabbit, 1:500; cat. no. ab31261, Abcam); Rab7 (rabbit, 1:3000; Gomez-Lamarca, et al., 2015); Rab11 (rabbit, 1:8000; Gomez-Lamarca et al., 2015). Phalloidin conjugated to FITC, Rhodamine and Alexa Fluor 647 (Life Technologies) was used at 1:50, 1:200 and 1:100, respectively, to image actin. Images were collected using an Olympus Fluoview 1000 confocal microscope with a 60×1.35 NA objective with ×1 or ×2 zoom, and measurements were taken with Fiji (http://fiji.sc/Fiji). Data was analysed and graphs produced using Microsoft Excel, Matlab and BoxPlotR (http://boxplot.tyerslab.com). For fluorescence intensity measurements, levels were modulated to avoid oversaturation, and the same laser intensities were used for each genotype or experimental condition. Aggregates and muscle defects were counted manually using Fiji.
